# Supplementary material for: Optimizing Antibiotic Prescribing for Acute Respiratory Tract Infections in German Primary Care: Results of the Regional Intervention Study CHANGE-3 and the Nested cRCT
Source: Antibiotics (Basel). 2023 May 4;12(5):850. doi: 10.3390/antibiotics12050850 (PMC10215067; doi:10.3390/antibiotics12050850)
Supplement: Supplementary file 1 [file antibiotics-12-00850-s001.zip › antibiotics-2355724-supplementary.pdf]

Supplementary Materials

Table S1: Data-based feedback for physicians.

1 Short Name	Data-based feedback for physicians
2 Goal and rationale	Self-reflection by means of practice-specific, data-based feedback reports and evidence-based background information
3 Materials	Feedback reports are based on the claims data (diagnosis and prescription data) of one's own practice; checklists, patient information
4 Procedures	Claims-data (§§295, 300 SGB V) are provided by the participating health insurance companies pseudonymized for practices and anonymised for patients. The patient, medical service and time filter was prepared together with the project partners (physicians, health insurance companies, evaluators, analysts) in advance. Participating practices have given informed consent in writing to use their practice specific data, which means that specific claims data can be allocated to each participating practice. A SFTP (Secure FILE Transfer Protocol) will be used for transmission of data between health insurance and the aQua Institute. Afterwards data will be checked and analysed using SPSS evaluates at the establishment number (BSNR) level.<br>Individualized reporting enables a self-developed software. The benchmark (mean 25% best practices) and control supply (comparison group) are shown for comparison purposes. The background information is guideline- and evidence-based (structured literature research).
5 Providers of intervention	Physicians
6 Mode of delivery	Individual intervention
7 Location	The materials were postally sent
8 Frequency	4th quarter of 2018, 2nd quarter of 2020
9 Planned tailoring	
10 Fidelity enhancement	

Table S2: Outreach education visit.

1 Short Name	Outreach education visit
2 Goal and rationale	Personal information
3 Materials	Slide presentation; tablets; flyers;
4 Procedures	During the visitation, practice-specific, organizational problems influencing the prescription of antibiotics are discussed with the entire practice team (e.g., time pressure / weekday-related fluctuations in the prescription of antibiotics / possible expectation of patients / "order prescriptions" / communication of symptomatic measures already at registration). In addition, the indicator-based, individual feedback reports received and returned by each intervention group practice are also reflected upon visitation. In addition, during the visitation, all available materials of the regional campaign (posters, information leaflets, information, short films, etc. aimed at the patients) will be specially introduced.
5 Providers of intervention	Trained visitor and practice team
6 Mode of delivery	On site visit, slide presentation and discussion
7 Location	Visitation in the practice
8 Frequency	Once in the 4th quarter of 2018 and 1rst quarter of 2019; duration approximately 1.5h, total of 55 intervention practices
9 Planned tailoring	
10 Fidelity enhancement	

Table S3: E-learning on communication with patients for physicians.

1 Short Name	E-learning for physicians to promote patient-centred communication.
2 Goal and rationale	The focus is on exploring patient expectations and shared decision-making in patients with non-complicated, acute infections, which are especially common in ambulatory practices and do not require the use of antibiotics as first choice treatment. The educational objective is to carry out a convincing physician-patient communication.
3 Materials	Video clips of re-enacted scenes in GP practices. Additional information as text/graphics.
4 Procedures	Various situations, typical for GP practices, are represented. In addition, the e-learning platform comprises background information, which can be displayed by the users, if required. GP practices will receive a user account for the whole practice team.
5 Providers of intervention	The screenplay is produced at the Institute of General Medicine of the University of Rostock and implemented by the Society for Patient-centered Communication mbH (GPZK) in collaboration with a director and actors. The e-learning platform is established at the aQua Institute with the help of an external service provider.
6 Mode of delivery	Single intervention<br>All physicians in the intervention group of CHANGE-3
7 Location	The content is integrated into a Learning Management System (LMS).<br>The learning platform will be accessible via the practice's user-portal. Access for users can be provided via the specific user-portal. The contents in the learning platform are secured. The learning contents are structured as self-learning courses. The learning platform will be available under the domain „welearn.academy".<br>The correct functioning of video contents is ensured by an early test. The learning contents are generated in HTML5 format using the SCORM standards.
8 Frequency	Each course lasts about 1 hour and can be completed in the 4th quarter of 2018, 1rst and 2nd quarter of 2019.
9 Planned tailoring	No
10 Fidelity enhancement	

Table S4: E-learning on communication with patients for non-physician health professionals of the practice team.

1 Short Name	E-learning for medical assistants to promote patient-centred communication.
2 Goal and rationale	The focus is on exploring patient expectations and shared decision-making in patients with non-complicated, acute infections, which are especially common in ambulatory practices and do not require the use of antibiotics as first choice treatment. The educational objective is to learn how to support the physician in their daily practice with patients with non-complicated infections and the rational use of antibiotics.
3 Materials	Video clips of re-enacted scenes of typical situations in the GP's daily practice, which are relevant for non-physician health professionals of the practice team.
4 Procedures	Various situations, typical for GP practices, are represented. In addition, the e-learning platform comprises background information of interest for medical assistants, which can be displayed by the users, if required.<br>The participation will be documented, and a personalized certificate will be sent out.
5 Providers of intervention	The screenplay is produced at the Institute of General Medicine of the University of Rostock (and, if possible, involving MFAs) and implemented by the Society for Patient-centered Communication mbH (GPZK) in collaboration with a director and actors. The e-learning platform is established at the aQua Institute with the help of an external service provider.
6 Mode of delivery	Single intervention<br>Tailored for non-physician health professionals from practice team in the intervention group of CHANGE-3
7 Location	The content is integrated into a Learning Management System (LMS).<br>The learning platform will be accessible via a user-portal. The contents on the learning platform are secured. Access for users can be provided via the practice's user-portal. The learning contents are structured as self-learning courses. The learning platform will be available under the domain „welearn.academy".<br>The correct functioning of video contents is ensured by an early test. The learning contents are generated in HTML5 format using the SCORM standards.
8 Frequency	Each course lasts about 1 hour and can be completed in the 4th quarter of 2018, 1rst and 2nd quarter of 2019.
9 Planned tailoring	No
10 Fidelity enhancement	

Table S5: Patient information material.

1 Short Name	Patient information material.
2 Goal and rationale	The information material focuses particularly on patients affected by infections and aims to raise awareness of „antibiotics / resistance development”.
3 Materials	Handout (guideline-based, culture-sensitive)
4 Procedures	The core messages are guideline-based and developed in agreement with the other consortium partners. The materials used are adapted materials from ARena (www.arena-info.de/patienten-info).
5 Providers of intervention	Physician, pharmacist, psychologist, journalist, advertising expert.
6 Mode of delivery	Group intervention<br>Some of the material is also available via the website “weniger-antibiotika.de”.
7 Location	Provision of material for the practices via the website “weniger-antibiotika.de”.
8 Frequency	as of 4th quarter 2018
9 Planned tailoring	
10 Fidelity enhancement	

Table S6: Information material available on tablet computers.

1 Short Name	Information material on tablets
2 Goal and rationale	The information material focuses particularly on patients affected by infections and aims to raise awareness of „antibiotics / resistance development”.
3 Materials	Topic-specific explanation videos and information on a tablet PC
4 Procedures	The core messages are guideline-based and developed in agreement with the other consortium partners. The materials used are adapted materials from the website “weniger-antibiotika.de” and adapted information for patients from ARena (www.arena-info.de/patienten-info).
5 Providers of intervention	Physician, pharmacist, psychologist, journalist, advertising expert, communication designers.
6 Mode of delivery	Group intervention<br>Some of the material is also available via the website “weniger-antibiotika.de”.
7 Location	In practices
8 Frequency	1rst quarter 2019 – 2nd quarter 2020
9 Planned tailoring	
10 Fidelity enhancement	
